# Quantum Contagion: A Quantum-Like Approach for the Analysis of Social Contagion Dynamics with Heterogeneous Adoption Thresholds

**DOI:** 10.3390/e23050538

**Published:** 2021-04-27

**Authors:** Ece C. Mutlu, Ozlem Ozmen Garibay

**Affiliations:** Department of Industrial Engineering and Management Systems, University of Central Florida, Orlando, FL 32816, USA

**Keywords:** complex networks, heterogeneous adoption thresholds, information diffusion, phase transitions, quantum-like social contagion, technology adoption

## Abstract

Modeling the information of social contagion processes has recently attracted a substantial amount of interest from researchers due to its wide applicability in network science, multi-agent-systems, information science, and marketing. Unlike in biological spreading, the existence of a reinforcement effect in social contagion necessitates considering the complexity of individuals in the systems. Although many studies acknowledged the heterogeneity of the individuals in their adoption of information, there are no studies that take into account the individuals’ uncertainty during their adoption decision-making. This resulted in less than optimal modeling of social contagion dynamics in the existence of phase transition in the final adoption size versus transmission probability. We employed the Inverse Born Problem (IBP) to represent probabilistic entities as complex probability amplitudes in edge-based compartmental theory, and demonstrated that our novel approach performs better in the prediction of social contagion dynamics through extensive simulations on random regular networks.

## 1. Introduction

Understanding and better modeling contagion dynamics in complex networks play a crucial role in shedding light on the spreading mechanisms of viral diseases, microfinance activities, information, harmful emotions, and technology adoptions. It not only gives us an opportunity to design more efficient anti-pathogen strategies during infectious disease outbreaks but also grants theoretical foundations to predict collective behaviors, and even mitigate the propagation of false information in social systems. Researchers have classified these spreading dynamics in different disciplines into two main categories: (i) biological, or (ii) social contagion. Despite the analogy between these spreading mechanisms, information (or behavioral) spreading has been found to have a distinct inherent characteristic, which is called social reinforcement effect [1,2,3], compared to biological spreading. The importance of the reinforcement effect in social contagion is that the simple contagion mechanism in epidemic spreading, which assumes that even one single activated source might be sufficient for the transmission, is transformed into a more complex contagion mechanism. This complexity in contagion dynamics is generally described by Markovian processes; these approaches are called threshold-driven, where the adoption occurs only in the existence of a certain fraction of neighbors who have already adopted, contrary to biological spreading. This significant effect in social contagion emphasizes the importance of network topology on the final adoption size and triggers discontinuous phase transitions in the final spreading size versus transmission probability. We can argue that possession of more complex dynamics than the largely examined epidemic contagion, and at the same time relating different disciplines such as marketing and information science, make understanding social contagion dynamics a substantial and unaccomplished task.

The pioneering study of Granovetter [4], in which a mathematical approach for social contagion is firstly introduced, proposed a linear threshold model based on the assumption that individuals’ behavior in a network can be affected by their neighbors’ actions. In this receiver-centric model, individuals adopt a behavior only if its certain fraction of neighbors have already adopted the behavior. Later, Goldenberg’s pioneering study [5] of diffusion in marketing became another well-known technique in social contagion studies. In this sender-centric model called the independent cascade model, each adopted node has a single chance to influence one of its susceptible neighbors. Recently, inspired by epidemic models, one of the most commonly used methods in the literature of social contagion studies is the message passing approach [6], in which individuals within the target population (or network) are divided into mutually exclusive compartments based on their current status and their future status at any time can be predicted based on the predefined rate of contact between compartments and their certain transition rates. As opposed to the conventional compartmental models, the reinforcement effect is also included with the existence of a threshold value for individuals to adopt the behavior. Therefore, the message passing approach is considered a non-Markovian process, which makes it more realistic in the application of real-world complex contagions.

The most challenging task in employing any of these approaches in social contagion analyses is to model the complexity of individuals. This complexity arises due to either the heterogeneity of the individuals in their adoption threshold or the uncertainty in their decision-making process during adoption. Although earlier studies employed a simplistic threshold model, i.e., uniform threshold distribution in social contagion studies, to address the former challenge, recent studies utilized more complex threshold distributions such as binary [3], tent-like function [7], truncated normal distribution function [8] or sigmoid function [9]. To the best of our knowledge, the uncertainty in their decision-making process has not been addressed yet in social contagion analyses despite its theoretical and experimental evidence in behavioral economics, decision science, cognitive science, or multi-agent systems. Although the whole process in social contagion studies is based on the assumption that individuals are perfectly rational and do follow the rules of classical probability theory and logic while taking an action during the process, it is well-known that only bounded rationality can exist [10] and individuals do not obey the classical probability rules [11,12,13,14]. It is mainly due to agent interactions through information exchange that can influence individuals’ emotions, change subconscious feelings, and trigger subjective biases [10,15]. Furthermore, the impacts of such behavioral effects become more significant when individuals make their decision under uncertainty [13]. To address this complexity in human decision-making and explain the corresponding irrationality and existing paradoxes and fallacies, researchers developed numerous quantum-like approaches [13,16,17,18,19,20]. Although classical approaches argue that human inference deterministically jumps between definite states across time, the main assumption behind quantum-like approaches is that competitive beliefs exist in the human mind at the same time. They form a composite entangled prospect for the decision-maker. Because behavior spreading in a social contagion is fueled with the successful transmission of behavior (or information) among two entangled binary prospects (adopting/not adopting) of decision-makers in a network, the utilization of these approaches in social contagion analyses may provide more realistic insights.

Social contagion is a prominent research area due to its wide applicability to different disciplines; therefore, it is highly studied in the existence of restricted contact [21], heterogeneous adoption threshold [3], local trend imitation [7], heterogeneous credibility [22] and with memory of non-redundant information [23]. All of these studies showed that these models are very effective in predicting social contagion dynamics within defined scenarios, except when transmission rates are close to the critical transmission probabilities. Because the phase transitions in the final adoption size pattern are commonly observed in the existence of individuals with a heterogeneous adoption threshold in the system, this phenomena demonstrates that classical approaches fall short in the modeling of social contagion dynamics in many cases. To address this problem, we believe that quantum-like approaches and interference effects leverage the extant social contagion analyses and better model its nonlinear dynamics even on critical transmission probabilities. To integrate a quantum-like approach, we employ the Inverse Born Problem (IBP), which argues that probabilistic entities can be represented by using complex probability amplitudes.

The rest of the paper is as follows: First, we explain the general social contagion mechanism that is used in this study and a methodology for the application of quantum-like edge-based compartmental theory. In the results section, we provide theoretical results of social contagion dynamics and numerical simulations on random regular networks with varying parameters.

## 2. Materials and Methods

In the context of network theory, a complex network, G〈V,E〉, is defined as the set of vertices (nodes) $\left(V=\left\{v_{1},v_{2},...,v_{n}\;|n\in N\right\}\right)$ and edges between them ($E_{v_{i},v_{j}}=\left(v_{i},v_{j}\right)$ where (i,j∈N;i≠j). To exemplify the social contagion mechanism in this study, we integrate a quantum-like point of view to the classical message-passing approach [6], which generalizes the well-known susceptible-adopted-recovered (SAR) model, to fully describe the mechanisms of information (or behavior) spreading on a complex network with *N* nodes and a degree distribution P(k). In this model, each individual in a network falls into one of three states: *susceptible*, *adopted* and *recovered*. An individual in a *susceptible* state (*S*) does not adopt the information yet. An *adopted* individual (*A*) adopts the information and tries to transmit it to each of its *susceptible* neighbors with a probability λ at each time step. After each successful transmission, the *susceptible* individual, who receives information from his *adopted* neighbor, updates his cumulative units of information, i.e., m⇒m+1. It should be noted that non-redundant, thus non-Markovian, information transmission is considered to focus on a more legit scenario, i.e., information can be transmitted only once from an *adopted* individual to a specific *susceptible* individual, who records each successful transmission at each time step. A *susceptible* individual becomes *adopted* if its cumulative units of information exceeds its threshold. Simultaneously, each adopted individual may lose his interest in the information and becomes *recovered* with a probability γ. Because *recovered* individuals will not further participate in information spreading, a steady-state is reached if all individuals in the network become *recovered* or there is no chance for individuals to change their current states. We initialize the social contagion model with a small fraction of individuals ($\rho_{0}$) assigned as *adopted* and the rest as *susceptible* in the network. In the rest of this study, S(t), A(t), and R(t) represent the fractions of susceptible, adopted, and recovered individuals at the time step *t*, respectively.

### 2.1. Preliminaries

Quantum approaches can facilitate modeling continuous state systems due to their more advanced representation compared to classical approaches. The classical approach uses set-theoretic representation and its sample space is defined as a set of possible events, for example, $\left\{m_{1},m_{2}\right\}$. On the other hand, the quantum approach uses vector space representation, and its sample space is a plane space spanned by the orthogonal basis vectors, for example, $|m_{1}\rangle$ and $|m_{2}\rangle$. A detailed explanation of differences between classical and quantum approaches and their applications is given in [16,17]. For more detailed information about the utilization of IBP in the quantum-like approach, please refer to Khrennikov’s quantum-like representation algorithm [24,25].

Because we aim to employ the quantum calculus of probability for our analysis, we draw from Born’s rule, which relates classical probability density function and a quantum probability amplitude by using wave function, and represent a classical probability as a squared magnitude of the complex amplitude (wave function). Therefore, the relation between a classical probability density function and a quantum probability amplitude is given by:

(1) $$Pr\left(A\right)={|e^{i\theta_{A}}\psi_{A}|}^{2}$$

Here, the exponential term ($e^{i\theta_{A}}$) is called the global phase factor of the quantum probability amplitude. The classical probability (Pr(A)) is related with a quantum probability amplitude ($e^{i\theta_{A}}\psi_{A}$), which corresponds to the amplitude of a wave function, and this relation to the classical probability is obtained by multiplying this amplitude with its complex conjugate, i.e., ${|e^{i\theta_{A}}\psi_{A}|}^{2}=e^{i\theta_{A}}\psi_{A}e^{-i\theta_{A}}\psi_{A}^{*}$. Although the result of an individual event probability in the classical probability theory converges to that in the quantum approach, the computation of the union of mutually exclusive events differ in these two methods. The quantum-like approach yields an extra term, *"interference effect"*, which does not exist in classical probability theory. To illustrate, suppose that we aim to obtain the union of three mutually exclusive events by using a classical probability formula, which is given by:

(2) Pr(A∪B∪C)=Pr(A)+Pr(B)+Pr(C)

The quantum counterpart of the classical probability of the union of three mutually exclusive events is obtained by using Born’s rule in Equation (1):

(3) $$\begin{array}{cc} Pr(A\cup B\cup C) & =|e^{i\theta_{A}}\psi_{A}+e^{i\theta_{B}}\psi_{B}+e^{i\theta_{C}}\psi_{C}{|}^{2}\\ \; & =e^{i\theta_{A}}\psi_{A}.e^{-i\theta_{A}}\psi_{A}+e^{i\theta_{A}}\psi_{A}.e^{-i\theta_{B}}\psi_{B}+e^{i\theta_{A}}\psi_{A}.e^{-i\theta_{C}}\psi_{C}\\ \; & +e^{i\theta_{B}}\psi_{B}.e^{-i\theta_{A}}\psi_{A}+e^{i\theta_{B}}\psi_{B}.e^{-i\theta_{B}}\psi_{B}+e^{i\theta_{B}}\psi_{B}.e^{-i\theta_{C}}\psi_{C}\\ \; & +e^{i\theta_{C}}\psi_{C}.e^{-i\theta_{A}}\psi_{A}+e^{i\theta_{C}}\psi_{C}.e^{-i\theta_{B}}\psi_{B}+e^{i\theta_{C}}\psi_{C}.e^{-i\theta_{C}}\psi_{C} \end{array}$$

Knowing that,

(4) $$cos\left(\theta_{1}-\theta_{2}\right)=\frac{e^{\theta_{1}-\theta_{2}}+e^{-\theta_{1}+\theta_{2}}}{2}$$

Equation (3) reduces to:

(5) $$\begin{array}{cc} Pr(A\cup B\cup C) & ={|\psi_{A}|}^{2}+{|\psi_{B}|}^{2}+{|\psi_{C}|}^{2}+2(|\psi_{A}\left|\right|\psi_{B}|cos\left(\theta_{A}-\theta_{B}\right)\\ & +|\psi_{A}\left|\right|\psi_{C}|cos\left(\theta_{A}-\theta_{C}\right)+|\psi_{B}\left|\right|\psi_{C}|cos\left(\theta_{B}-\theta_{C}\right)) \end{array}$$

The additional terms in Equation (5) compared to Equation (2) are called *"interference terms"*, which does not exist in classical probability theory [19,24,25,26].

### 2.2. Edge-Based Compartmental Theory

Inspired by numerous studies [3,7,22], we employ an edge-based compartmental theory to understand the dynamics of the quantum social contagion approach. Suppose that u,(u∈V) is an individual who is in a susceptible state, i.e., he can receive information from his neighbors but cannot transfer since he has not adopted information yet. Let v,(v∈V) be a randomly chosen neighbor of *u* ($E_{u,v}\neq 0$). If we define θ(t) as the probability that the individual *v* has not transmitted information to an individual *u* by time *t*, the probability that individual *u* with degree $k_{u}$ has received *m* pieces information from his distinct neighbors by time *t* will be binomially distributed and expressed as:

(6) τm(ku,t)=kumθ(t)(ku−m)(1−θ(t))m

The quantum counterpart of this step is intuitively the same, because the binomial distribution property holds true (please refer to Appendix A.1 for the mathematical proof). If the individual *u* could receive enough pieces of information from his distinct neighbors to exceed his threshold ($\phi_{u}$), i.e., $m\geq \phi_{u}$, he will adopt the information and try to transmit it to his susceptible neighbors in the next time step. Otherwise, he will keep his susceptible state in the next time step. Thus, the probability of individual *u* with degree $k_{u}$ being susceptible is:

(7) $$\begin{array}{cc} s_{u}\left(k_{u},t\right) & =\sum_{\phi_{u}}F\left(\phi_{u}\right)\sum_{m=0}^{\phi_{u}-1}\tau_{m}\left(k_{u},t\right) \end{array}$$

where $F(\phi_{u})$ denotes the information adoption threshold function. The quantum-like social contagion is a novel approach, and it introduces a complexity via its additional interference terms. Because the heterogeneity of individuals in information adoption is significant, we assume that $F(\phi_{u})$ can be represented as a binomial distribution. In other words, individuals may have either a relatively lower threshold ($T_{A}=1$) with probability *p*, or a relatively higher threshold ($T_{B}>1$) with probability 1−p. Thus;

(8) $$F\left(\phi_{u}\right)=\left\{\begin{array}{cc} T_{A}, & \mathrm{with}\;\mathrm{probability}\;p\\ T_{B}, & \mathrm{with}\;\mathrm{probability}\;1-p \end{array}\right.$$

We obtain the fraction of susceptible individuals at time *t* by combining Equations (7) and (8) with the degree distribution of the network as:

(9) S(t)=∑kuP(ku)su(ku,t)=∑kuP(ku)pθ(t)ku+(1−p)∑m=0TB−1kumθ(t)(ku−m)(1−θ(t))m

We can follow a similar strategy to calculate the probability of individual *v* with degree $k_{v}$ being a susceptible state. Being in a susceptible state, the individual *u* is unable to transmit the information to its neighbor *v*. Thus, the individual *v* can receive information from his $k_{v}-1$ distinct neighbors. Taking all possible values of receiving *m* pieces of cumulative information and $\phi_{v}$ into consideration, we obtain:

(10) sv(kv,t)=pθ(t)(kv−1)+(1−p)∑m=0TB−1kvmθ(t)(kv−m−1)(1−θ(t))m

Recall that the transfer between states of individuals occurs not only between susceptible and adopted states but also adopted and recovered states. Adopted individuals may lose their interest in the transmission process and move into the recovered state with a predefined probability. Thus, the following set of ordinary differential equations (ODEs) define the time dependence of the individuals in each compartment in the system described above.

(11) $$\begin{array}{cc} \frac{dA(t)}{dt} & =-\frac{dS(t)}{dt}-\gamma A\left(t\right)\\ \frac{dR(t)}{dt} & =\gamma A(t) \end{array}$$

By computing θ(t), we can solve the equations for S(t), and also A(t) and R(t), and investigate the system dynamics. In edge-based compartmental theory, we have not made any assumption about the state of individual *v*; therefore, θ(t) may consist of three possible outcomes that are mutually exclusive in the classical approach:

(12) $$\theta \left(t\right)=\xi_{S}\left(t\right)+\xi_{A}\left(t\right)+\xi_{R}\left(t\right)$$

where $\xi_{S}\left(t\right)$ ($\xi_{A}\left(t\right)$, $\xi_{R}\left(t\right)$) represents the probability that a neighbor *v* is in a susceptible (adopted, recovered) state and has not transmitted the information to individual *u* by time *t*.

To employ quantum probability rules, we can use Born’s rule in Equation (1) and write the counterpart of Equation (12) as follows as in Equation (5):

(13) $$\begin{array}{cc} \theta (t) & ={|e^{i\theta \psi_{\xi_{S}\left(t\right)}}+e^{i\theta \psi_{\xi_{A}\left(t\right)}}+e^{i\theta \psi_{\xi_{R}\left(t\right)}}|}^{2}\\ & ={|\psi_{\xi_{S}\left(t\right)}|}^{2}+{|\psi_{\xi_{A}\left(t\right)}|}^{2}+{|\psi_{\xi_{R}\left(t\right)}|}^{2}+2[|\psi_{\xi_{S}\left(t\right)}\left|\right|\psi_{\xi_{A}\left(t\right)}|cos\left(\theta_{\xi_{S}\left(t\right)}-\theta_{\xi_{A}\left(t\right)}\right)\\ & +|\psi_{\xi_{S}\left(t\right)}\left|\right|\psi_{\xi_{R}\left(t\right)}|cos\left(\theta_{\xi_{S}\left(t\right)}-\theta_{\xi_{R}\left(t\right)}\right)+|\psi_{\xi_{A}\left(t\right)}\left|\right|\psi_{\xi_{R}\left(t\right)}|cos\left(\theta_{\xi_{A}\left(t\right)}-\theta_{\xi_{R}\left(t\right)}\right)] \end{array}$$

Here, the amplitude ${|\psi_{\xi_{S}\left(t\right)}|}^{2}$ refers to $P(\xi_{S}\left(t\right))$, ${|\psi_{\xi_{A}\left(t\right)}|}^{2}$ to $P(\xi_{A}\left(t\right))$ and ${|\psi_{\xi_{R}\left(t\right)}|}^{2}$ to $P(\xi_{R}\left(t\right))$. The angle $\theta_{\xi_{S}\left(t\right)}-\theta_{\xi_{A}\left(t\right)}$ corresponds to the phase of the inner product between $|\xi_{S}\left(t\right)|$ and $|\xi_{A}\left(t\right)|$. Note that there is no direct transition from the susceptible state to recovered state, so $cos(\theta_{\xi_{S}\left(t\right)}-\theta_{\xi_{R}\left(t\right)})$ will be equal to 0. By recalling inverse Born’s rule again, we can finalize the relation above as:

(14) $$\begin{array}{cc} \theta (t) & =\xi_{S}\left(t\right)+\xi_{A}\left(t\right)+\xi_{R}\left(t\right)\\ & +\sqrt{\xi_{S}\left(t\right)\xi_{A}\left(t\right)}cos\left(\theta_{\xi_{S}\left(t\right)}-\theta_{\xi_{A}\left(t\right)}\right)+\sqrt{\xi_{A}\left(t\right)\xi_{R}\left(t\right)}cos\left(\theta_{\xi_{A}\left(t\right)}-\theta_{\xi_{R}\left(t\right)}\right) \end{array}$$

Herein, the additional terms are called interference terms that do not exist in classical probability theory. From this point, we will call $\sqrt{\xi_{S}\left(t\right)\xi_{A}\left(t\right)}cos\left(\theta_{\xi_{S}\left(t\right)}-\theta_{\xi_{A}\left(t\right)}\right)$ SA interference term and $\sqrt{\xi_{A}\left(t\right)\xi_{R}\left(t\right)}cos\left(\theta_{\xi_{A}\left(t\right)}-\theta_{\xi_{R}\left(t\right)}\right)$ as AR interference term for the sake of simplicity.

Later, we draw from statistical network science to make the connection between these two individuals *u* and *v*. In the case of the existence of an uncorrelated network, the probability of an edge connecting individual *v* with a degree $k_{v}$ to one of its neighbors, for example, individual *u* with degree $k_{u}$, is equal to $k_{v}P\left(k_{v}\right)/\langle k\rangle$, where 〈k〉 is the mean degree. Thus, it can be obtained that:

(15) $$\begin{array}{cc} \xi_{S}\left(t\right) & =\frac{\sum_{k_{v}}k_{v}P\left(k_{v}\right)s_{v}\left(k_{v},t\right)}{\langle k\rangle } \end{array}$$

θ(t) is a time-dependent variable, and it will not accomplish its definition after any successful transmission. Therefore, we need to consider its time-dependence to fully understand the systems’ dynamics from the beginning till the steady-state. If we suppose that an adopted individual transmits behavioral information with probability λ, the decrease in θ(t) can be written as:

(16) $$\frac{d\theta (t)}{dt}=-\lambda \xi_{A}\left(t\right)$$

At time *t*, the behavioral information is not transmitted with probability 1−λ and the adopted individuals move into a recovered state with probability γ, simultaneously. Then;

(17) $$\frac{d\xi_{R}\left(t\right)}{dt}=\gamma \left(1-\lambda \right)\xi_{A}\left(t\right)$$

Substituting Equation (16) into (17) and integrating it with the initial conditions of θ(0)=1 and $\xi_{R}\left(0\right)=0$, we can obtain:

(18) $$\xi_{R}\left(t\right)=\frac{\gamma (1-\lambda )[1-\theta (t)]}{\lambda }$$

Finally, we obtain $\xi_{A}\left(t\right)$ inserting Equations (15) and (18) into (13) by using a computational knowledge engine. Substituting the resulting equation of $\xi_{A}\left(t\right)$ into Equation (16), we derive the time evolution of θ(t). For further details, please refer to Appendix A.2. Furthermore, the dynamics of quantum social contagion can be described with the ODE equations in Equation (11). When t→∞, we find the final adoption size R(∞) once the degree distribution is known.

## 3. Results

**Figure 1:**
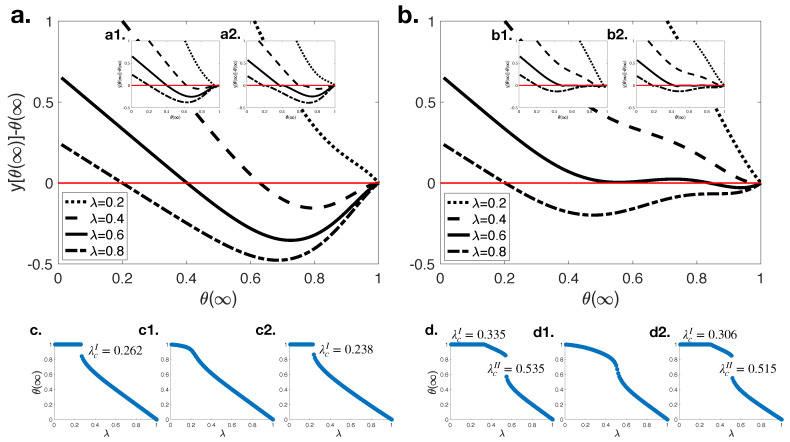
The graphical solution of the fixed point of equation dθ(t)/dt at steady-state when (**a**) $T_{b}=2$ and (**b**) $T_{b}=4$ on random regular networks (〈k〉=10 and p=0.3). The physically meaningful solutions of θ(t) for each possible λ values are shown when (**c**) $T_{b}=2$ and (**d**) $T_{b}=4$. Subplots (**a1**, **b1**,**c1**,**d1**) show the relative solutions when $cos(\theta_{\xi_{S}\left(t\right)}-\theta_{\xi_{A}\left(t\right)})=0.2$, while subplots (**a2**,**b2**,**c2**,**d2**) do when $cos(\theta_{\xi_{A}\left(t\right)}-\theta_{\xi_{R}\left(t\right)})=0.2$.

In this study, our main goal is to compare and contrast the dynamics and performances of classical social contagion along with its quantum counterpart. The differences in these two approaches stem from the definition of θ(t) in Equations (12) and (14); therefore, we first investigated the graphical solution of the fixed point equation dθ(t)/dt at steady-state, i.e., t→∞ with different threshold values on random regular networks in Figure 1.

Figure 1a shows the graphical solution of a fixed point of equation dθ(t)/dt when $T_{b}=2$. Results show that there is only one nontrivial solution when λ is small; however, at moderate λ values there are cases in which two nontrivial solutions are observed. In such a case, only the maximum solution is physically meaningful. In Figure 1c, we plotted the physically meaningful solutions of θ(t) for each possible λ values by using the classical approach. The solution for θ(∞) shows a discontinuous change and jump to another point at critical transmission probability ($\lambda_{c}^{I}=0.262$). Therefore, R(∞) grows discontinuously with the increasing λ. The quantum approach, on the other hand, yields two interference terms: SA ($cos(\theta_{\xi_{S}\left(t\right)}-\theta_{\xi_{A}\left(t\right)})$) and AR ($cos(\theta_{\xi_{S}\left(t\right)}-\theta_{\xi_{A}\left(t\right)})$) interference terms. Figure 1a(a1,a2) shows the graphical solution of fixed point of equation dθ(t)/dt at steady-state when $T_{b}=2$ and only SA (AR) interference is observed. The change in θ(∞) with respect to λ in the existence of SA (AR) interference is also plotted in Figure 1c(c1,c2). Results show that the SA interference term makes the pattern continuous, i.e., R(∞) increases continuously with the increasing λ. In the existence of the AR interference term, on the other hand, the same discontinuous change pattern is observed as in classical social contagion, however, at lower critical transmission probability ($\lambda_{c}^{I}=0.238$).

For the case of $T_{b}=4$ (Figure 1b), θ(∞) decreases continuously and a continuous phase transition is observed at the first critical transmission probability ($\lambda_{c}^{I}=0.335$), then another discontinuous change occurs at the second critical transmission probability ($\lambda_{c}^{II}=0.535$) in the classical approach (Figure 1d). This means that R(∞) first increases continuously and then a discontinuous pattern is observed, which is called a hybrid phase transition. W. Wang et al. [3] explain this situation as follows: In the existence of more than one critical transmission probability, two different types of information adoption occur: (i) local adoption in which the information is adopted by a small fraction of individuals, (ii) global adoption in which the information is adopted by a finite fraction of individuals. When $\lambda <\lambda_{c}^{I}$, individuals adopt information locally, while a global adoption is observed when $\lambda >\lambda_{c}^{II}$. In the case of $\lambda_{c}^{I}<\lambda <\lambda_{c}^{II}$, individuals who have lower thresholds adopt behavior globally while individuals who have higher thresholds adopt behavior locally. In the quantum social contagion model, the SA interference term makes R(∞) growth continuous with increasing λ, while the AR term displays same pattern as in the classical approach with lower the critical transmission probabilities, i.e., ($\lambda_{c}^{I}=0.306$ and $\lambda_{c}^{II}=0.515$).

**Figure 2:**
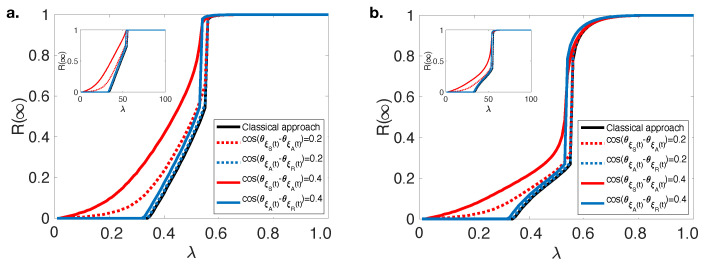
The final information adoption size R(∞) versus λ with classical (black solid line), various $cos(\theta_{\xi_{S}\left(t\right)}-\theta_{\xi_{A}\left(t\right)})=0.2$ and 0.4 (red dash and solid lines) and $cos(\theta_{\xi_{A}\left(t\right)}-\theta_{\xi_{R}\left(t\right)})=0.2$ and 0.4 (blue dash and solid lines) when (**a**) $T_{b}=2$ and (**b**) $T_{b}=4$ on random regular networks (〈k〉=10 and p=0.3). Subplots show the same simulations when 〈k〉=15.

Figure 2 shows R(∞) versus λ by using the classical approach and varying strength of SA and AR interference terms in Equation (14) when $T_{b}=2$ and $T_{b}=4$. As we mentioned, classical approaches show a hybrid phase transition in both cases. This hybrid phase transition pattern is also observed when only AR interference exists; however, a second-order (continuous) phase transition is observed in the existence of SA interference. We observed the similar pattern until $T_{b}\geq 6$ only, because after this level the fraction of individuals who have a lower adoption threshold were not enough to persuade individuals who have higher adoption threshold in the system. Furthermore, the phase transition becomes continuous even in the classical approach also when $T_{b}=1$, because the model reduces to the traditional SAR model [1]. The mini subplots on the left-top corner of each figure shows same dynamics when the mean-degree (〈k〉) of RNN is increased, and same conclusions are observed. Therefore, we can conclude that our results are robust to the changes in 〈k〉 of RNNs.

**Figure 3:**
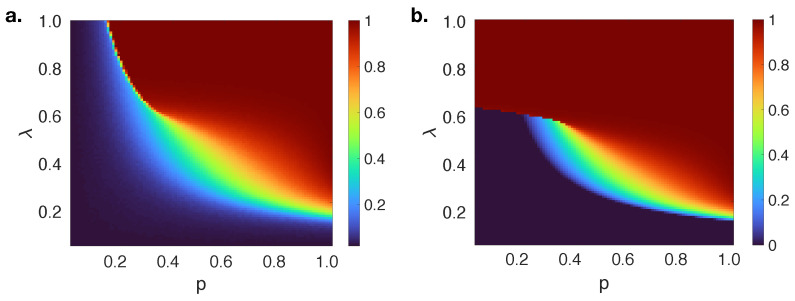
The dependence of R(∞) on *p* and λ on random regular networks with 〈k〉=10 and $T_{b}=4$ as a result of (**a**) numerical simulations, (**b**) theoretical analysis by using a classical approach.

For the comparison of performances of classical and quantum approaches in this study, extensive numerical simulations are performed on uncorrelated random regular networks (RRNs) with N=10,000, 〈k〉=10 and γ=1.0. Figure 3 shows the fraction of adopted individuals with varying behavioral information transmission probability (λ) and initial fraction of adopted individuals (*p*). The theoretical solutions of R(∞) described in Figure 1 can be seen at the λp plane more clearly. A first continuous, then a discontinuous increase in R(∞) shows a hybrid phase transition. This crossover phenomena in the increase of R(∞) with respect to *p* separates the λp plane into three different regions: (i) region I (p≤0.15), only a negligibly small fraction of individuals adopt the information (local information adoption), (ii) region II with a first-order phase transition (0.15<p≤0.42), a definite fraction of individuals adopt the information above $\lambda_{c}^{I}$, (iii) region III with a second order phase transition (p>0.42), a global adoption is observed above $\lambda_{c}^{I}$. On the other hand, a theoretical analysis by using a classical approach fails to model the spreading mechanism in region I when *p* is small, because θ(∞) is observed to be equal to one until a fixed value; although, it has a gradually decreasing pattern in numerical simulations. This results in an overestimation of final adoption size in this region. Moreover, a smooth transition of R(∞) on the λp plane in region II in numerical simulations is also modeled with a redundant sharp transition in the classical approach.

**Figure 4:**
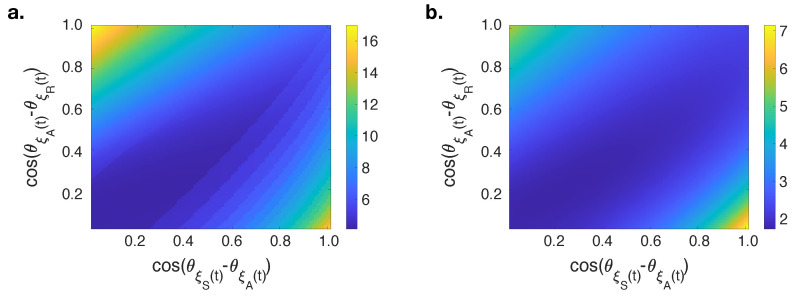
The dependence of error between R(∞) numerical simulations and theoretical analysis by using a quantum-like approach on $cos(\theta_{\xi_{S}\left(t\right)}-\theta_{\xi_{A}\left(t\right)})$ and $cos(\theta_{\xi_{A}\left(t\right)}-\theta_{\xi_{R}\left(t\right)})$ interference terms when (**a**) p=0.3, (**b**) p=0.6.

The squared difference of R(∞) versus λ from 0.01 to 1.00 (0.01 increments) between results obtained via theoretical analysis and numerical simulations using a quantum-like approach with varying interference terms are shown in Figure 4. The origin point represents the squared error when the classical approach is used because both interference terms are equal to zero ($e_{p=0.3}^{2}=4.1707$ and $e_{p=0.6}^{2}=1.7643$). Regardless of the initial fraction of adopted individuals (*p*), the minimum errors are observed near to the diagonal of interference terms plane, and the minimum value is obtained when $cos(\theta_{\xi_{S}\left(t\right)}-\theta_{\xi_{A}\left(t\right)})=0.15$ and $cos(\theta_{\xi_{A}\left(t\right)}-\theta_{\xi_{R}\left(t\right)})=0.16$ ($e_{p=0.3}^{2}=4.1229$ and $e_{p=0.6}^{2}=1.7326$). These results demonstrate that the quantum-like approach in an edge-based compartmental model of a message passing approach in the modeling of social contagion performs better compared to the classical method because it can better predict the final adoption size close to the critical transmission probabilities.

## 4. Discussion

The spread of ideas, attitudes, or behavioral patterns among a group of individuals is called social contagion. Although this spread among individuals used to be regarded as a pathogen in a biological spreading, empirical studies demonstrated that social contagion is far more complex due to the social, cognitive, and behavioral differences of humans. The complexity of humans in a social contagion process is addressed by considering the heterogeneity of their adoption thresholds with the assumption of perfect rationality; however, numerous empirical studies demonstrate that humans violate the rules of classical probability while making decisions. In order to improve the modeling of human decision-making about the adoption of information and/or behavior, we employed a quantum-like approach in social contagion analysis as well as assigning individuals heterogeneous adoption thresholds. We believe that our method, so-called quantum contagion, is able to portray the complexity of individuals and better model a social contagion process. We integrate an Inverse Born Problem (IBP) to represent classical probabilistic entities as complex probability amplitudes in a quantum-like message-passing approach. An edge-based compartmental theory is used to quantify the classical and quantum theoretical models, and a large number of simulations on RRNs are carried out for the comparison of their performances. In this study, we employed a two-state spreading threshold model in which individuals have a relatively low threshold ($T_{A}=1$) with probability *p*, and a relatively high threshold ($T_{B}>1$) with probability 1−p. The effect of threshold heterogeneity with varying network properties has been already investigated in previous studies. These studies showed that two different types of information adoption occur in the existence of more than one critical transmission probability: local and global adoption. The local adoption is observed when $\lambda <\lambda_{c}^{I}$ and information is adopted by a small fraction of individuals who have a lower adoption threshold. Whereas, the global adoption occurs when $\lambda >\lambda_{c}^{II}$ and information is adopted by a finite fraction of individuals. In the case of $\lambda_{c}^{I}<\lambda <\lambda_{c}^{II}$, individuals who have lower thresholds adopt behavior globally, while individuals who have higher thresholds adopt behavior locally. Although edge-based compartmental theory can model social contagion dynamics in most cases, these analyses fall short when transmission rates are close to these critical transmission probabilities. In the classical social contagion model, the final adoption size (R(∞)) grows discontinuously with the increasing behavioral information transmission probability (λ). Numerical investigations carried out on RRNs show that the quantum social contagion model performs better than the extant classical social contagion model because it is able to model the dynamics near the critical transmission probabilities. The quantum social contagion model displays the same hybrid phase transition pattern; however, both phase transitions are observed at lower critical transmission probability values. It means that local and global adoption behavior in the two-state spreading threshold model is observed earlier than the classical approach. The sharp discontinuous changes in final adoption size near the critical transmission probabilities are also observed as smoother in the quantum approach. Testing our conclusions on different mean degrees of RNN and with a different initial fraction of adopted individuals also demonstrates the generalizability and robustness of our conclusions. The optimum SA and AR interference values remain the same, as the initial fraction of adopted individuals changes. Thus, we argue that interference in quantum social contagion is not dependent on the initial fraction of adopted individuals on the network. Future studies may aim to find a heuristic to predict interference effects in the quantum approach to model social contagion dynamics without a calibration. Moreover, the effects of varying network topology on quantum social contagion dynamics remain open for further exploration. We intend to continue our analyses in these directions. It should be noted that despite the quantum-like approach in edge-based compartmental theory bringing heterogeneity due to the entangled structure of complex amplitudes in λ, we assumed that each adopted node has an equal chance to transmit the behavior to his susceptible neighbors. Thus, we ignored the influence variety of specific nodes in the spreading mechanism. Researchers can integrate IBP to other high performance theoretical approaches for epidemic spreading such as dynamical message passing and/or edge-based mean-field theory; however, these techniques yield very complex equations, and the quantum-like approach may exacerbate its complexity to make this problem even more challenging to resolve [27].

## Data Availability

Not applicable.

## Appendix A. Mathematical Proofs of Quantum Counterpart of Equations

### Appendix A.1. Quantum Counterpart of Equation (6)

Since we have defined θ(t) as the probability that the individual *v* has not transmitted information to an individual *u* by time *t*, the quantum probability of the same event can be calculated by using Born’s rule in Equation (1) as follows:

(A1) $$|\sqrt{\theta (t)}e^{\theta (t)}{|}^{2}=\left(\sqrt{\theta (t)}e^{\theta (t)}\right).\left(\sqrt{\theta (t)}e^{-\theta (t)}\right)=\theta \left(t\right)$$

Similarly, the probability of a successful transmission is equal to:

(A2) $$\begin{array}{cc} |\sqrt{1-\theta (t)}e^{\left(1-\theta (t)\right)}{|}^{2}\\ \; & =\left(\sqrt{1-\theta (t)}e^{\left(1-\theta (t)\right)}\right).\left(\sqrt{1-\theta (t)}e^{\left(\theta (t)-1\right)}\right)=1-\theta \left(t\right) \end{array}$$

Therefore, we get exactly the same outcome as in the classical theory.

(A3) τm(ku,t)=kumθ(t)(ku−m)(1−θ(t))m

### Appendix A.2. Calculation of ξ_A_(t) and Time Evolution of θ(t) in Classical and Quantum Approach

Inserting Equations (15) and (18) into (12) yields $\xi_{A}\left(t\right)$ by using the classical approach and gives the following expression:

(A4) $$\begin{array}{cc} \xi_{A}\left(t\right) & =\theta \left(t\right)-\frac{\sum_{k_{v}}k_{v}P\left(k_{v}\right)s_{v}\left(k_{v},t\right)}{\langle k\rangle }-\frac{\gamma (1-\lambda )[1-\theta (t)]}{\lambda } \end{array}$$

Substituting Equation (A4) into (16) gives us the time evolution of theta(t) as:

(A5) $$\frac{d\theta (t)}{dt}=-\lambda \left[\theta \left(t\right)-\frac{\sum_{k_{v}}k_{v}P\left(k_{v}\right)s_{v}\left(k_{v},t\right)}{\langle k\rangle }\right]-\gamma \left(1-\lambda \right)\left[1-\theta \left(t\right)\right]$$

The quantum counterpart of this calculation, however, is more complex due to the interference terms in Equation (14). In this case, we insert Equations (15) and (18) into (14) to obtain $\xi_{A}\left(t\right)$ by using the quantum approach:

(A6) $$\begin{array}{cc} \theta (t) & =\frac{\sum_{k_{v}}k_{v}P\left(k_{v}\right)s_{v}\left(k_{v},t\right)}{\langle k\rangle }+\xi_{A}\left(t\right)+\frac{\gamma (1-\lambda )[1-\theta (t)]}{\lambda }\\ & +\sqrt{\frac{\sum_{k_{v}}k_{v}P\left(k_{v}\right)s_{v}\left(k_{v},t\right)}{\langle k\rangle }\xi_{A}\left(t\right)}cos\left(\theta_{\xi_{S}\left(t\right)}-\theta_{\xi_{A}\left(t\right)}\right)\\ & +\sqrt{\xi_{A}\left(t\right)\frac{\gamma (1-\lambda )[1-\theta (t)]}{\lambda }}cos\left(\theta_{\xi_{A}\left(t\right)}-\theta_{\xi_{R}\left(t\right)}\right)\lambda ) \end{array}$$

Solving this quadratic equation for $\xi_{A}\left(t\right)$ yields four different roots; the physically meaningful one is as follows:

(A7) $$\begin{array}{cc} \; & \xi_{A}\left(t\right)=\frac{1}{2}[cos^{2}\left(\theta_{\xi_{S}\left(t\right)}-\theta_{\xi_{A}\left(t\right)}\right)\frac{\sum_{k_{v}}k_{v}P\left(k_{v}\right)s_{v}\left(k_{v},t\right)}{\langle k\rangle }\\ \; & -\sqrt{}(cos^{4}\left(\theta_{\xi_{S}\left(t\right)}-\theta_{\xi_{A}\left(t\right)}\right){\left(\frac{\sum_{k_{v}}k_{v}P\left(k_{v}\right)s_{v}\left(k_{v},t\right)}{\langle k\rangle }\right)}^{2}\\ \; & -4cos^{3}\left(\theta_{\xi_{S}\left(t\right)}-\theta_{\xi_{A}\left(t\right)}\right)cos(\theta_{\xi_{A}\left(t\right)}-\theta_{\xi_{R}\left(t\right)}){\left(\frac{\sum_{k_{v}}k_{v}P\left(k_{v}\right)s_{v}\left(k_{v},t\right)}{\langle k\rangle }\right)}^{\left(3/2\right)}\sqrt{}(\frac{\gamma (1-\lambda )[1-\theta (t)]}{\lambda })\\ \; & +6cos^{2}\left(\theta_{\xi_{S}\left(t\right)}-\theta_{\xi_{A}\left(t\right)}\right)cos^{2}\left(\theta_{\xi_{A}\left(t\right)}-\theta_{\xi_{R}\left(t\right)}\right)\frac{\sum_{k_{v}}k_{v}P\left(k_{v}\right)s_{v}\left(k_{v},t\right)}{\langle k\rangle }\frac{\gamma (1-\lambda )[1-\theta (t)]}{\lambda }\\ \; & +4cos^{2}\left(\theta_{\xi_{S}\left(t\right)}-\theta_{\xi_{A}\left(t\right)}\right)\theta \left(t\right)\frac{\sum_{k_{v}}k_{v}P\left(k_{v}\right)s_{v}\left(k_{v},t\right)}{\langle k\rangle }\\ \; & -4cos^{2}\left(\theta_{\xi_{S}\left(t\right)}-\theta_{\xi_{A}\left(t\right)}\right){\left(\frac{\sum_{k_{v}}k_{v}P\left(k_{v}\right)s_{v}\left(k_{v},t\right)}{\langle k\rangle }\right)}^{2}\\ \; & -4cos^{2}\left(\theta_{\xi_{S}\left(t\right)}-\theta_{\xi_{A}\left(t\right)}\right)\frac{\sum_{k_{v}}k_{v}P\left(k_{v}\right)s_{v}\left(k_{v},t\right)}{\langle k\rangle }\frac{\gamma (1-\lambda )[1-\theta (t)]}{\lambda }\\ \; & -4cos(\theta_{\xi_{S}\left(t\right)}-\theta_{\xi_{A}\left(t\right)})cos^{3}\left(\theta_{\xi_{A}\left(t\right)}-\theta_{\xi_{R}\left(t\right)}\right)\sqrt{}(\frac{\sum_{k_{v}}k_{v}P\left(k_{v}\right)s_{v}\left(k_{v},t\right)}{\langle k\rangle }){\left(\frac{\gamma (1-\lambda )[1-\theta (t)]}{\lambda }\right)}^{\left(3/2\right)}\\ \; & -8cos(\theta_{\xi_{S}\left(t\right)}-\theta_{\xi_{A}\left(t\right)})cos(\theta_{\xi_{A}\left(t\right)}-\theta_{\xi_{R}\left(t\right)})\theta \left(t\right)\sqrt{}(\frac{\sum_{k_{v}}k_{v}P\left(k_{v}\right)s_{v}\left(k_{v},t\right)}{\langle k\rangle })\sqrt{}(\frac{\gamma (1-\lambda )[1-\theta (t)]}{\lambda })\\ \; & +8cos(\theta_{\xi_{S}\left(t\right)}-\theta_{\xi_{A}\left(t\right)})cos(\theta_{\xi_{A}\left(t\right)}-\theta_{\xi_{R}\left(t\right)}){\left(\frac{\sum_{k_{v}}k_{v}P\left(k_{v}\right)s_{v}\left(k_{v},t\right)}{\langle k\rangle }\right)}^{\left(3/2\right)}\sqrt{}(\frac{\gamma (1-\lambda )[1-\theta (t)]}{\lambda })\\ \; & +8cos(\theta_{\xi_{S}\left(t\right)}-\theta_{\xi_{A}\left(t\right)})cos(\theta_{\xi_{A}\left(t\right)}-\theta_{\xi_{R}\left(t\right)})\sqrt{}(\frac{\sum_{k_{v}}k_{v}P\left(k_{v}\right)s_{v}\left(k_{v},t\right)}{\langle k\rangle }){\left(\frac{\gamma (1-\lambda )[1-\theta (t)]}{\lambda }\right)}^{\left(3/2\right)}\\ \; & +cos^{4}\left(\theta_{\xi_{A}\left(t\right)}-\theta_{\xi_{R}\left(t\right)}\right){\left(\frac{\gamma (1-\lambda )[1-\theta (t)]}{\lambda }\right)}^{2}+4cos(\theta_{\xi_{A}\left(t\right)}-\theta_{\xi_{R}\left(t\right)})\theta \left(t\right)\left(\frac{\gamma (1-\lambda )[1-\theta (t)]}{\lambda }\right)\\ \; & -4cos^{2}\left(\theta_{\xi_{A}\left(t\right)}-\theta_{\xi_{R}\left(t\right)}\right)\left(\frac{\sum_{k_{v}}k_{v}P\left(k_{v}\right)s_{v}\left(k_{v},t\right)}{\langle k\rangle }\right)\left(\frac{\gamma (1-\lambda )[1-\theta (t)]}{\lambda }\right)\\ \; & -4^{2}cos(\theta_{\xi_{A}\left(t\right)}-\theta_{\xi_{R}\left(t\right)}){\left(\frac{\gamma (1-\lambda )[1-\theta (t)]}{\lambda }\right)}^{2})\\ \; & -2cos(\theta_{\xi_{S}\left(t\right)}-\theta_{\xi_{A}\left(t\right)})cos(\theta_{\xi_{A}\left(t\right)}-\theta_{\xi_{R}\left(t\right)})\sqrt{}(\frac{\sum_{k_{v}}k_{v}P\left(k_{v}\right)s_{v}\left(k_{v},t\right)}{\langle k\rangle })\sqrt{}(\frac{\gamma (1-\lambda )[1-\theta (t)]}{\lambda })\\ \; & +cos^{2}\left(\theta_{\xi_{A}\left(t\right)}-\theta_{\xi_{R}\left(t\right)}\right)\left(\frac{\gamma (1-\lambda )[1-\theta (t)]}{\lambda }\right)+2\theta \left(t\right)-2\left(\frac{\sum_{k_{v}}k_{v}P\left(k_{v}\right)s_{v}\left(k_{v},t\right)}{\langle k\rangle }\right)\\ \; & -2\left(\frac{\gamma (1-\lambda )[1-\theta (t)]}{\lambda }\right)] \end{array}$$
